# Screening of Reference Genes for Quantitative Real-Time PCR Analysis in Tissues and during Testis Development, and Application to Analyze the Expression of *kifc1* in *Hemibarbus labeo* (Teleostei, Cypriniformes, Cyprinidae)

**DOI:** 10.3390/ani14132006

**Published:** 2024-07-07

**Authors:** Xinming Gao, Siqi Liu, Yaoping Lv, Qingmin Dai, Ling Zhu, Zehui Hu, Junkai Lu, Haidong Zhou, Jing Jin

**Affiliations:** 1 College of Ecology, Lishui University, Lishui 323000, China; nbugxm4851@163.com (X.G.); liusiqi020522@163.com (S.L.); sophie128341@126.com (Q.D.); zhuling202306@163.com (L.Z.); 2 Zhejiang Marine Fisheries Research Institute, Zhoushan 316100, China; deg813@126.com; 3 Cixi Fisheries Technology Extension Center, Ningbo 315300, China; lujunkai2023@126.com; 4 Suichang Fisheries and Agricultural Machinery Technology Extension Station, Lishui 323399, China; lssczhd@163.com; 5 Zhejiang Fisheries Technology Extension Center, Hangzhou 311100, China; 18892626457@163.com

**Keywords:** quantitative real-time PCR, *Hemibarbus labeo*, reference gene, testis development, *kifc1*

## Abstract

**Simple Summary:**

Quantitative real-time PCR technology is widely employed in the detection of gene expression levels in animals. In this technique, the selection of reference genes directly impacts the accuracy of the results. Currently, there is a lack of studies addressing suitable reference genes for *Hemibarbus labeo*, an economically important freshwater fish in China. In this study, we evaluated the expression stability of nine housekeeping genes in tissues and during testis development to ascertain appropriate reference genes. We found that *eef1a* exhibits high expression stability in tissues and during testis development. Therefore, it is suitable as a reference gene for quantitative real-time PCR analysis. Furthermore, we assessed the optimal number of reference genes needed when calculating gene expression levels using the geomean method, a method for analyzing gene expression using multiple reference genes. We found that two reference genes suffice for precise quantification. Additionally, we utilized the selected reference genes to analyze the expression of *kifc1*, a kinesin gene, to analyze its function in spermatogenesis. This constitutes the pioneering effort in identifying suitable reference genes for quantitative real-time PCR studies in *H. labeo*, thereby laying the groundwork for future explorations into gene expression regulation in this species.

**Abstract:**

The selection of proper reference genes is vital for ensuring precise quantitative real-time PCR (qPCR) assays. This study evaluates the stability of the expression of nine candidate reference genes in different tissues and during testicular development in *H. labeo*. The results show that *eef1a* is recommended as a reference gene for qPCR analysis in tissues and during testicular development. Furthermore, we evaluated the optimal number of reference genes needed when calculating gene expression levels using the geomean method, revealing that two reference genes are sufficient. Specifically, *eef1a* and *rps27* are recommended for analysis of gene expression in tissues, whereas *eef1a* and *actb* are advised for evaluating gene expression during testicular development. In addition, we examined the expression pattern of *kifc1*, a kinesin involved in the reshaping of spermatids. We detected peak expression levels of *kifc1* in testes, with its expression initially increasing before decreasing throughout testicular development. The highest expression of *kifc1* was observed in stage IV testes, the active period of spermiogenesis, suggesting a possible role for *kifc*1 in the regulation of the reshaping of spermatids and hence testicular development. This study represents the first investigation of reference genes for *H. labeo*, providing a foundation for studying gene expression patterns and investigating gene expression regulation during testicular development.

## 1. Introduction

Gene expression studies are important in molecular biology and genetics to understand the functional roles that genes play within an organism. In this field, the quantitative real-time PCR (qPCR) technique is a powerful tool and has been widely used to analyze the gene expression of transcription levels because of its great sensitivity, rapidity, accuracy, and reproducibility [1,2]. However, the results of qPCR can be affected by some factors, including the purity, concentration, and integrality of RNA and cDNA, and the amplification efficiency of the primer [1,3]. Therefore, in pursuit of precise and dependable outcomes from qPCR analyses, the employment of one or multiple reference genes is indispensable for effectively calibrating and normalizing the qPCR results. Housekeeping genes, including *actb* and *gapdh*, are often used as a reference gene [3]. However, studies have shown that the expression of some housekeeping genes is not static and is not suitable as reference gene in different tissues, different physiological traits, and different stages of development of the organism [4,5,6,7]. Therefore, it is necessary to select suitable internal reference genes with stable expressions when studying the gene expression of targeted genes under specific conditions or particular developmental stages.

Spermatogenesis is an important part of the study of animal reproductive biology, which involves the proliferation and differentiation of spermatogonia, the meiosis of spermatocyte, and the spermiogenesis, a process of spermatid development into sperm [8]. In fish, testis development is categorized into VI distinct stages, primarily determined by variations in the composition of germ cells [9,10]. The progression from stage I to V in fish testes represents a maturation continuum, culminating in the degenerative phase characterized by stage VI testes [9,10]. The spermatogenesis of most fish occurs within the spermatogenic cysts, culminating in the release of mature spermatozoa into the lobular lumen or tubule lumen [11,12]. A noteworthy feature in most fish spermiogenesis is the absence of acrosome formation [11,12], a stark contrast to mammalian spermiogenesis. Spermatogenesis is a complex process tightly regulated by many genes [13,14,15]. Screening for genes that regulate spermatogenesis is beneficial for revealing the mechanism of spermatogenesis and facilitating the regulation of animal reproduction. Therefore, it is of great significance for artificial animal reproduction in animal husbandry. In the study of animal spermatogenesis, it is very important to use qPCR to analyze the expression of genes of interest in tissues and during testis development [16,17]. However, several studies have shown that some housekeeping genes are not suitable as reference genes in gonadal development [4,18,19]. Therefore, it is important to select suitable reference genes for qPCR analysis in tissues and during testes development to analyze the function of genes in spermatogenesis.

*Hemibarbus labeo* belongs to the order Cypriniformes and the family Cyprinidae. It is an economically important freshwater fish in China, with delicious taste characteristics and rich in nutrition. Previous research on *H. labeo* has concentrated on diverse aspects, including the nutritional composition of its muscle [20], growth performance characteristics [21,22], the mechanisms underlying intermuscular bone formation [23], molecular immunology [24], and explorations in population genetics [25,26]. However, studies on the reference gene for qPCR and the regulatory mechanism of spermatogenesis have not been reported, which is detrimental to the molecular biology and reproduction biology research of *H. labeo*. Considering the significance of understanding testicular development and spermatogenesis mechanisms for reproductive biology and the advancement of artificial breeding techniques, it is essential to identify suitable reference genes during testicular development in *H. labeo*.

In this study, therefore, we screen the reference genes for qPCR analysis in tissues and during testes development. Furthermore, we analyze the expression of a kinesin gene *kifc1* in different tissues and during testis development to analyze its function in the testes and spermatogenesis. This study provides an important reference for the molecular biology study, particularly involving qPCR analysis, and contributes significantly to understanding the function of *kifc1* in spermatogenesis in *H. labeo*.

## 2. Materials and Methods

### 2.1. Sample Collection

*H. labeo* was sampled from the Jinman Aquatic Seedling Farm in Lishui, China. From February 2023 to July 2023, sixteen fish were collected every month. The gonads were extracted to identify the sex and then the male testes were divided into two portions. One portion was placed in an enzyme-free centrifuge tube (Axygen, Hangzhou, China) and immediately in liquid nitrogen for mRNA extraction, and the other was fixed in Bouin’s solution (Scientific phygene, Fuzhou, China) for 24 h for paraffin embedding to analyze the testicular development stage. In addition, the testes, intestines, livers, spleens, hearts, gills, kidneys, brains, and muscles were extracted from five adult male fish in April. These tissues were placed in enzyme-free centrifuge tubes and immediately frozen in liquid nitrogen and subsequently stored at −80 °C until mRNA extraction.

### 2.2. Identification of Testis Development

The development of the testes was analyzed using the tissue section technique. Identification of the development stage of the testes referred to Fu et al. [9] and Wang et al. [10]. First, fresh testes were fixed in Bouin’s solution for 24 h at room temperature, and then transferred to a 70% ethanol solution (Sinopharm, Shanghai, China) until tissues were dehydrated in alcohol and dimethylbenzene (Sinopharm, Shanghai, China) and embedded in paraffin blocks. The protocol for tissue embedding involved the following steps: Initially, tissues were sequentially dehydrated in 70%, 80%, and 90% ethanol solutions, each for 45 min. This was followed by two rounds of dehydration in 95% ethanol, for 35 min each. Subsequently, tissues underwent two 20 min immersions in 100% ethanol. Next, they were immersed in a 1:1 mixture of ethanol and xylene for 20 min, a process repeated twice. Afterward, tissues were subjected to three 20 min incubations in pure xylene. Then, they were infiltrated in a 1:1 ethanol–xylene solution for 1 h at 65 °C. This was succeeded by a 2 h infiltration in pure paraffin at the same temperature, followed by an additional 1 h infiltration in paraffin. Finally, the tissues were embedded in paraffin, which, upon cooling and solidification, formed paraffin blocks. The blocks were cut into 7 µm thick sections. After dewaxing with dimethylbenzene, the sections were stained with hematoxylin and eosin (H.E) staining kit (Beyotime, Shanghai, China) according to the manufacturer’s instructions. After mounting and drying, the sections were observed and photographed using a NI-U light microscope (Nikon, Tokyo, Japan).

### 2.3. RNA Extraction and Reverse Transcription

TRIzol reagent (Cwbio, Beijing, China) was used to extract total RNA from tissues, including the intestines, livers, spleens, hearts, gills, kidneys, brains, muscles, and testes in different stages of development according to the manufacturer’s instructions. RNA concentration was determined using a Nano-100 micro-spectrophotometer (Allsheng, Hangzhou, China) and its integrity was assessed by electrophoresis on 1.0% agarose gel. Good-quality RNA was used as a template for reverse transcription to synthesize cDNA using the StarScript II first-strand cDNA synthesis kit with gDNA remover (GenStar, Beijing, China).

### 2.4. Primer Design, Validation, and PCR Amplification Efficiency

The cDNA sequence of candidate reference genes (*gapdh*, *actb*, *tubb4b*, *eef1α*, *rpl7l1*, *rps27*, *rps4*, *18s*, *snrpd1*) is derived from our transcriptome sequencing data (data were not published). Their cDNA sequences have already been uploaded to the National Center for Biotechnology Information (https://www.ncbi.nlm.nih.gov; accessed on 21 March 2024), and their accession numbers are shown in Table 1. The primers were designed using Primer Premier 6.0 software (Table 2). The specificity of the primers was analyzed using PCR and quantitative real-time PCR (the method is described below). Only primers that produced a single amplification product as well as the solubility curve with individual peak were used for subsequent experiments (Figure S1). None of these primers detected an amplification product in the no-template controls lacking cDNA. Furthermore, the PCR products were analyzed by bidirectional sequencing (Youkang, Hangzhou, China) to further confirm the specificity of the primers. The PCR amplification efficiency of the primers was also analyzed through quantitative real-time PCR. The amplification efficiency (E value) was calculated with the formula E = (10^−1/slope^ − 1) × 100%.

### 2.5. Quantitative Real-Time PCR

The expression of nine candidate reference genes and *kifc1* was assayed by quantitative real-time PCR (qPCR). The qPCR reaction system included 10 μL of Master Mix (GenStar, Beijing, China), 5 μL of 1: 90 diluted cDNA, 0.3–1 μL of each primer (the primer concentration is 10 μM, and their dosage is shown in Table 2), and 3–4.4 μL of PCR-grade water (the total volume of primers and PCR grade water is 5 μL). The reactions were carried out and monitored in a LightCycler 480 system (Roche, Basel, Switzerland) with the following conditions: 95 °C for 3 min; 40 cycles of 95 °C for 15 s; 60 °C for 8 s; and 72 °C for 8 s. Three technical replicates were performed in this experiment.

Relative expression levels of the *kifc1* were calculated using the 2^−∆∆Ct^ method for a single reference gene [27] and using the geomean method for multiple reference genes [28]. The testes were used to determine the relative expression of *kifc1* in different tissues. Stage IV testes were used to determine the relative expression of *kifc1* during testes development. Each experiment analyzed gene expression in four separate biological replicates for robust statistics. Data were presented as means ± standard deviation (SD; *n* = 4).

### 2.6. Data Analysis

The evaluation of reference genes was carried out using the online tools RefFinder (http://blooge.cn/RefFinder/; accessed on 5 January 2024) [29] and GeNorm (https://seqyuan.shinyapps.io/seqyuan_prosper/; accessed on 5 January 2024) [30]. Upon acquiring the data through the aforementioned online tools, we subsequently utilized the Origin 2021 software (OriginLab, Northampton, MA, USA) to create figures. The significance of differences in *kifc1* gene expression in different tissues and different stages of testes development was analyzed using SPSS 21.0 (SPSS, Inc., Chicago, IL, USA). For normally distributed data sets with homogeneity of variance, the significance of the differences was analyzed by one-way analysis of variance (ANOVA). Otherwise, the significance of the differences was analyzed using a nonparametric test. Data with *p* < 0.05 were considered significant.

## 3. Results

### 3.1. Primer Specificity and Amplification Efficiency

The specificity of primers was verified by gel electrophoresis and sequencing, and their melting curves were monitored during qPCR experiments. As shown in Figure S1, a single amplicon was detected (Figure S1A), which upon bidirectional sequencing matched the sequence of the target gene. In the qPCR experiments, all melting curves exhibited single peaks (Figure S1B), indicating that the designed primers specifically amplify the target gene and are therefore specific. Furthermore, the amplification efficiency of nine candidate reference genes and *kifc1* was 94.8–108.7%. The correlation coefficients (R^2^ values) fell between 0.990 and 1.000 (Table 2). These results demonstrate the effectiveness of primers for qPCR experiment.

### 3.2. Cycle Threshold (Ct) Values of Candidate Reference Genes in Tissues and during Testis Development

**Figure 1:**
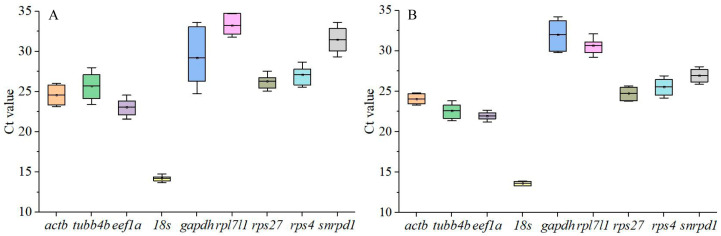
Expression levels of eight candidate reference genes in tissues (**A**) and during testis development (**B**) as the Ct mean. The line across the box is the mean. The boxes indicate the 25/75 percentiles. The whisker caps indicate the standard deviation.

In all tissues and testes at different stages of development, the Ct values ranged between 13.12 and 35. The *18s* gene had the smallest Ct value, ranging between 13.29 and 15.75 in different tissues, and ranging between 13.12 and 14.13 during testis development (Figure 1). Conversely, the *rpl7l1* gene showed the highest Ct values across different tissues, varying from 29.68 to 35; the *gapdh* gene had the highest Ct values during testis development, oscillating between 28.35 and 34.65 (Figure 1).

### 3.3. Stability of Expression of Candidate Reference Genes in Tissues in Adult Male Fish

**Figure 2:**
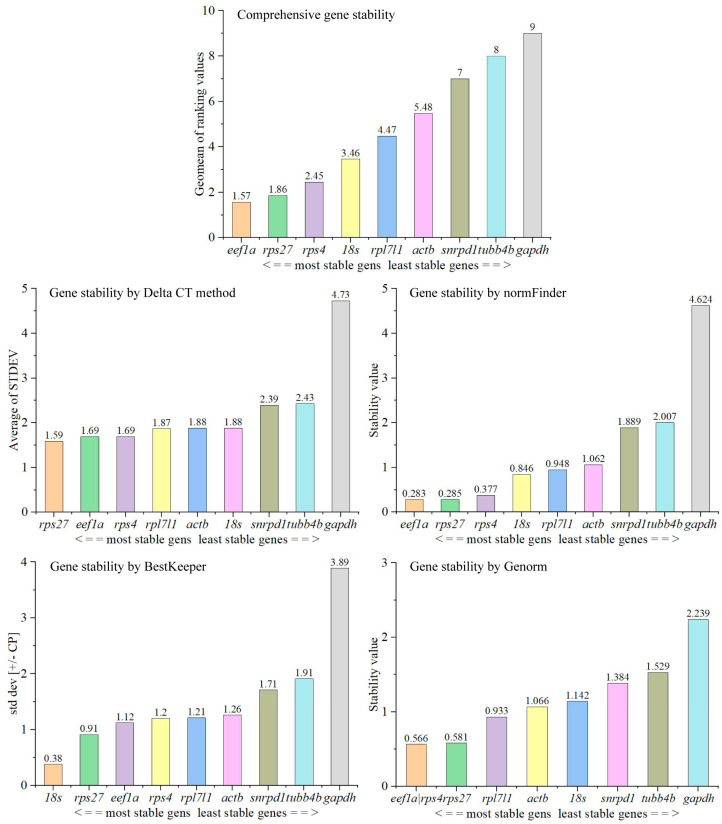
Stability of expression of candidate reference genes in tissues.

The gene most stably expressed is *rps27* using the Delta CT method, *eef1a* by normFinder, *18s* by BestKeeper (Figure 2). Both *eef1a* and *rps4* are the genes that are expressed more stably by Genorm analyzation (Figure 2). The RefFinder is a comprehensive analysis tool to calculate the stability of gene expression. Through the analysis of RefFinder, we found that the comprehensive gene stability of the nine candidate genes was sorted as *eef1a* > *rps27* > *sps4* > *18s* > *rpl7l1l* > *actb* > *snrpd1* > *tubb4b* > *gapdh* in tissues (Figure 2). Considering the stability of expression among the candidate reference genes, *eef1a* is recommended as the preferred reference gene for quantitative PCR (qPCR) analyses in tissues, employing the 2^−ΔΔCt^ method. The *rps27* gene ranks next in suitability for this purpose.

### 3.4. Stability of Expression of Candidate Reference Genes during Testes Development

#### 3.4.1. Identification of the Developmental Stage of the Testes

**Figure 3:**
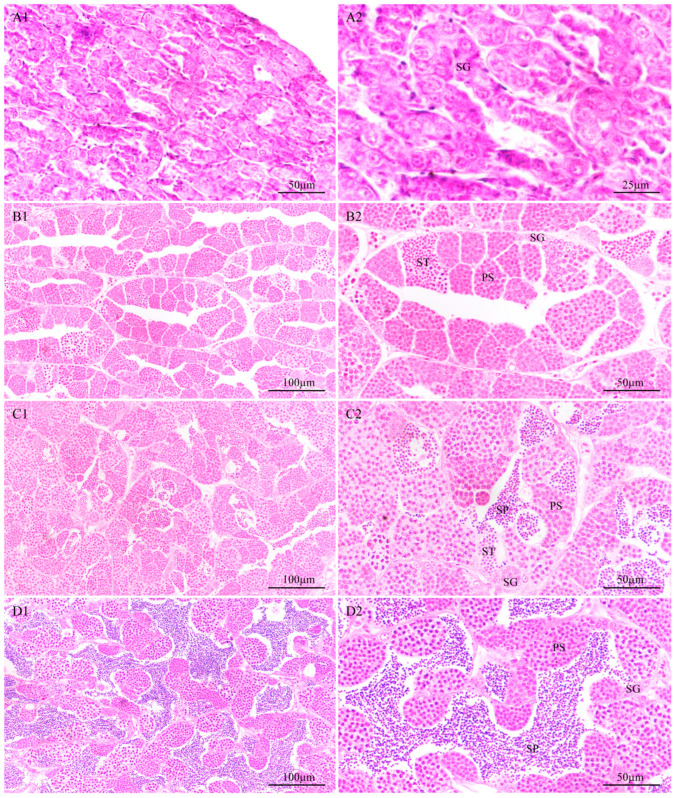
Identification and features of testes in different stages of development. (**A1**,**A2**) Testis at Stage II. In this stage, the testes are mainly composed of spermatogonia. (**B1**,**B2**) Testis at Stage III. In this stage, a large number of spermatogonia have been differentiated into spermatocytes. Meanwhile, a small number of spermatids have been observed. (**C1**,**C2**) Testis at Stage IV. This stage is a period of active spermiogenesis, and a large number of spermatids are developing into sperm. (**D1**,**D2**) Testis at Stage V. Large amounts of sperm are present in the testis in this stage. SG: spermatogonia; PS: primary spermatocyte; ST: spermatid; SP: sperm.

In our sampling, we obtained stage II to stage V testes. In stage II testes, they are mainly composed of spermatogonia and seminiferous lobules have been clearly observed (Figure 3(A1,A2)). In stage III testes, massive differentiation of spermatogonia into spermatocytes resulted in the testes being dominated by spermatocytes. Meanwhile, small amounts of spermatids can be observed (Figure 3(B1,B2)). In stage IV testes, some spermatids have differentiated and developed into sperm (Figure 3(C1,C2)), which are released into the lobular lumen. This stage is the most active period of spermiogenesis. In stage V testes, a large amount of sperm have been formed, which are full of the lobular lumen (Figure 3(D1,D2)).

#### 3.4.2. Selection of Reference Genes for qPCR Analysis during Testicular Development

**Figure 4:**
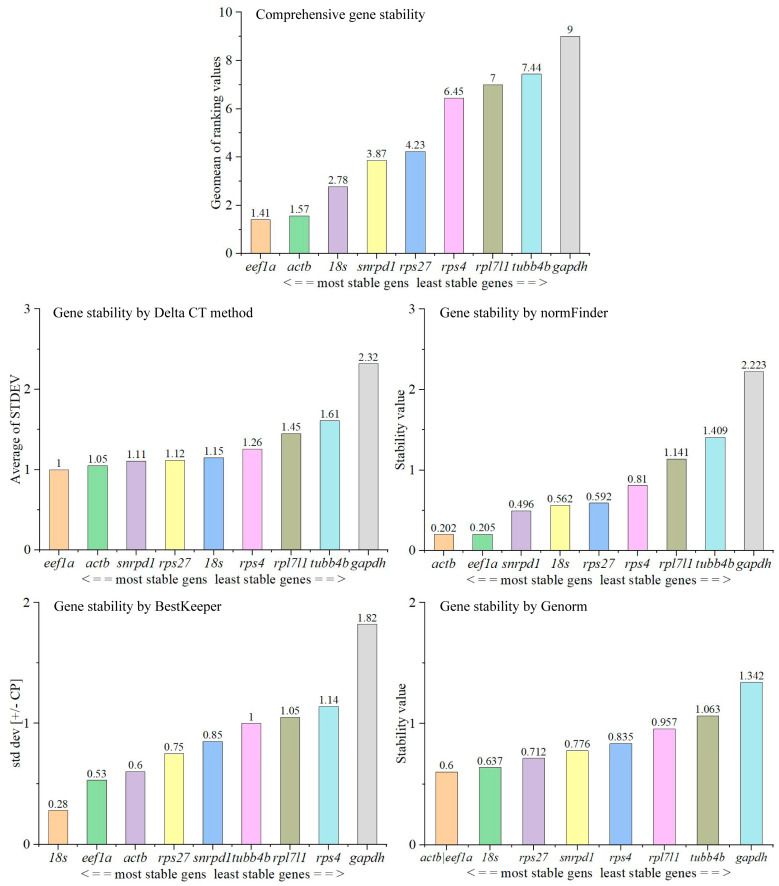
Stability of expression of candidate reference genes during testes development.

The gene most stably expressed is *eef1a* using the Delta CT method, *actb* using normFinder, *18s* using BestKeeper (Figure 4). Both *eef1a* and *actb* are the genes that are more stably expressed by Genorm analyzation (Figure 4). The comprehensive gene stability of nine candidate genes was sorted as *eef1a* > *actb* > *18s* > *snrpd1* > *rps27* > *rps4* > *rpl7l1* > *tubb4b* > *gapdh* during testis development (Figure 4). Therefore, *eef1a* is recommended as a reference gene during testicular development for qPCR using 2^−∆∆Ct^ method. This was followed by *actb*.

### 3.5. Optimal Number of Reference Genes Required for Quantification in Tissues and during Testis Development

**Figure 5:**
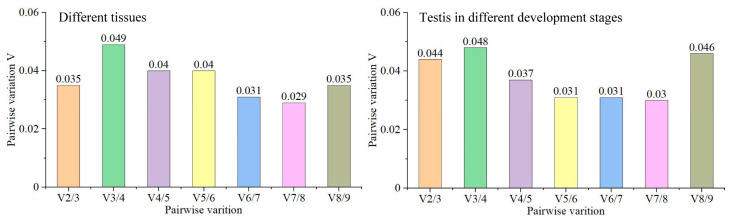
Optimal number of reference genes required for quantification in tissues and during testis development.

In the qPCR experiment, multiple reference genes were suggested to accurately calculate the expression level of a target gene by the geomean method. Therefore, we analyze the appropriate number of reference genes for quantification in tissues and during testicular development using the pairwise variation value (V) using geNorm. The V value is recommended to be less than 0.15 to determine the appropriate number of reference genes. Interestingly, we found that the pairwise variation values of V 2/3, V 3/4, V 4/5, V 5/6, V 6/7, V 7/8 and V 8/9 were less than 0.15 (Figure 5). Taking into account the experimental cost, workload, and stability of gene expression, we recommend *eef1a* and *rps27* as reference genes to calibrate the expression levels of target genes in tissues using the geomean method, and *eef1a* and *actb* as reference genes to calibrate the expression levels of target genes during testicular development.

### 3.6. Expression of kifc1 in Different Tissues

**Figure 6:**
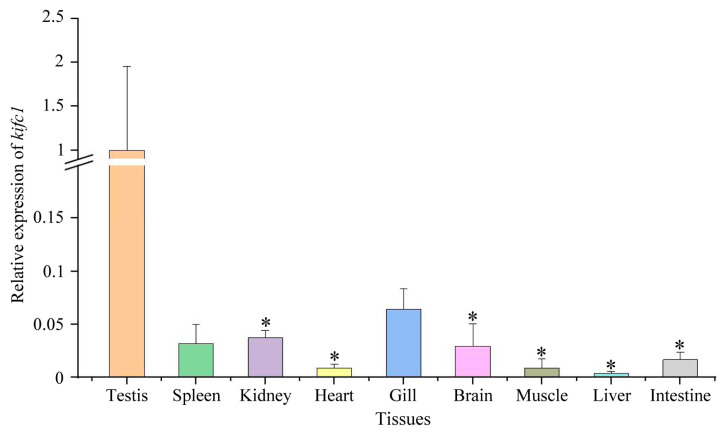
Expression of *kifc1* in different tissues. The expression of *kifc1* was calculated with *eef1a* and *rps27* together as reference genes. The highest expression of *kifc1* was observed in the testes. The asterisks above the column indicate a statistically significant difference (*p* < 0.05) in the expression of *kifc1* compared to that in the testis. Each experiment analyzed gene expression in four separate biological replicates. All values are the mean ± standard deviation (*n* = 4).

We analyze *kifc1* expression in different tissues using *eef1a* and *rps27* as reference genes. The results showed that *kifc1* was expressed in the testis, spleen, kidney, heart, gill, brain, muscle, liver, and intestine (Figure 6). Additionally, the expression of *kifc1* in the testes was higher than in other tissues (Figure 6), implying that *kifc1* may play an important role in the testes.

**Figure 7:**
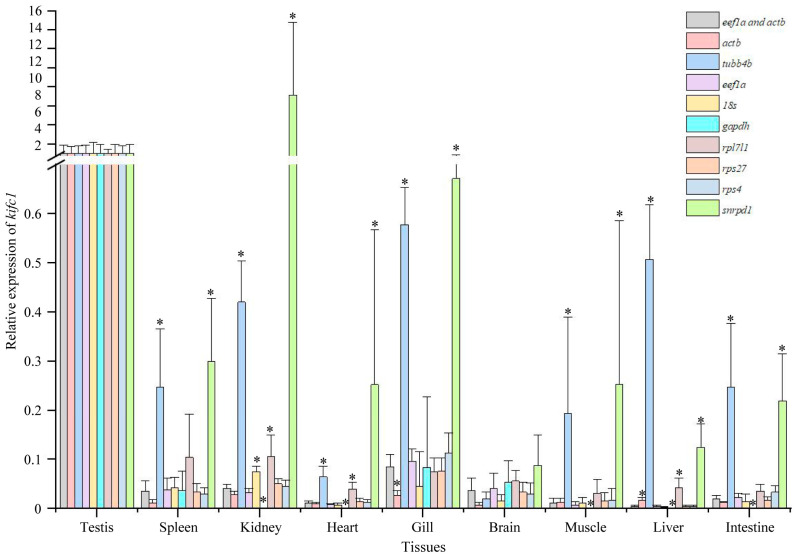
Expression of *kifc1* in different tissues with *eef1a* and *rps27* together as reference genes and with *actb*, *tubb4b*, *eef1a*, *18s*, *gapdh*, *rpl7l1*, *rps27*, *sps4*, and *snrpd1* as reference gene, respectively. The asterisks above the column indicate that the results are statistically significantly different (*p* < 0.05) when compared to using *eef1a* and *rps27* together as reference genes. Each experiment analyzed gene expression in four separate biological replicates. All values are the mean ± standard deviation (*n* = 4).

We also analyzed the expression of *kifc1* with *actb*, *tubb4b*, *eef1a*, *18s*, *gapdh*, *rpl7l1*, *rps27*, *sps4*, and *snrpd1* as reference genes, respectively. We found that the expression of *kifc1* was highest in the testes with *actb*, *tubb4b*, *eef1a*, *18s*, *gapdh*, *rpl7l1*, *rps27*, or *rps4* as reference genes, except *snrpd1* (Figure 7). However, unlike the results obtained using multiple reference genes or when *eef1a* serves as the reference gene, using *tubb4b*, *rpl7l1*, and *snrpd1* as the reference gene leads to an overestimated relative expression of *kifc1* in some tissues, meaning that the detected relative expression level is higher than its actual expression (Figure 7). Conversely, using *gapdh* as the reference gene results in an underestimated relative expression of *kifc1* in some tissues, indicating that the measured level is lower than its true expression (Figure 7). These findings suggest that using *tbbb4b*, *rpl7l1*, *snrpd1*, or *gapdh* as reference genes can produce inaccurate qPCR results in tissues.

### 3.7. Expression of kifc1 during Testis Development

**Figure 8:**
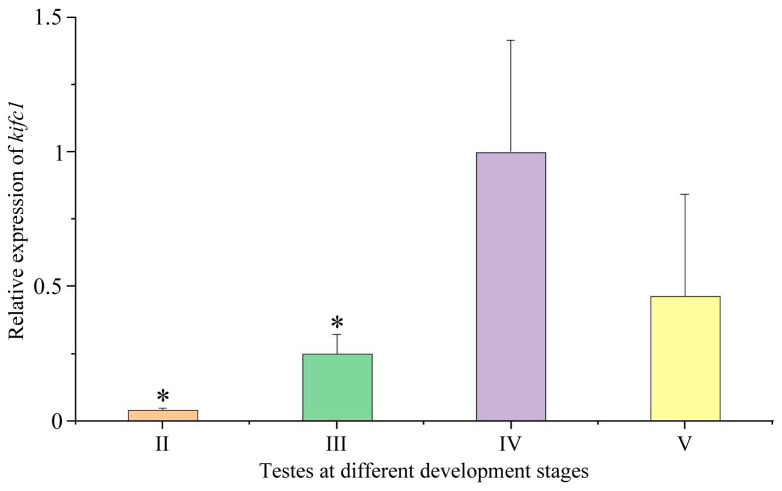
Expression of *kifc1* during testes development. The expression of *kifc1* was calculated with *eef1a* and *rps27* together as reference genes. The highest expression of *kifc1* was observed in stage IV testes. The asterisks above the column indicate a statistically significant difference (*p* < 0.05) in the expression of *kifc1* compared to that in the stage IV testes. Each experiment analyzed gene expression in four separate biological replicates. All values are the mean ± standard deviation (*n* = 4). II: stage II testes; III: stage III testes; IV: stage IV testes; V: stage V testes.

We analyzed *kifc1* expression during testis development with *eef1a* and *rps27* together as reference genes. The *kifc1* was consistently expressed during testis development. Its expression first increased and then decreased with the development of the testes (Figure 8). The expression of *kifc1* in stage IV testes was higher than in other stages (Figure 8), suggesting its important role in this stage.

**Figure 9:**
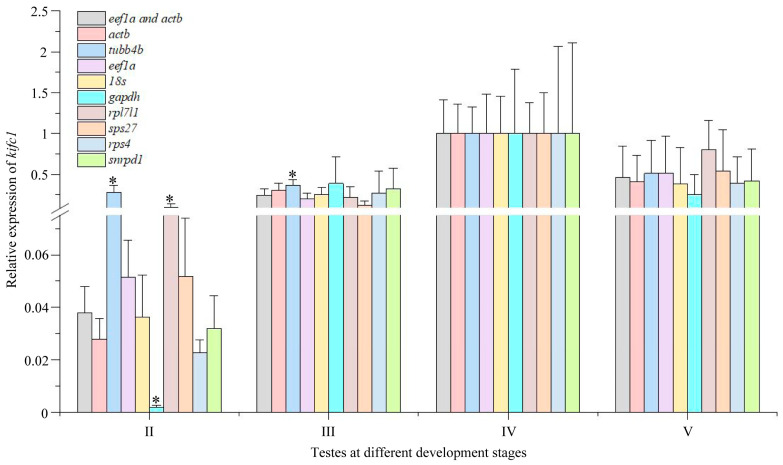
Expression of *kifc1* in different tissues with *eef1a* and *rps27* together as reference genes and with *actb*, *tubb4b*, *eef1a*, *18s*, *gapdh*, *rpl7l1*, *rps27*, *sps4*, and *snrpd1* as reference gene, respectively. The asterisks above the column indicate that the results are statistically significantly different (*p* < 0.05) when compared to using *eef1a* and *rps27* together as reference genes. Each experiment analyzed gene expression in four separate biological replicates. All values are the mean ± standard deviation (*n* = 4). II: stage II testes; III: stage III testes; IV: stage IV testes; V: stage V testes.

Furthermore, we analyzed the expression of *kifc1* during testis development with *actb*, *tubb4b*, *eef1a*, *18s*, *gapdh*, *rpl7l1*, *rps27*, *sps4*, and *snrpd1* as reference genes, respectively. The results indicate that the expression of *kifc1* first increased and then decreased with the development of testes, with the highest expression in the stage IV testes (Figure 9). However, in contrast to results obtained using multiple reference genes or those based on *actb*, *eef1a*, *18s*, *rps27*, *sps4*, and *snrpd1* for the analysis of *kifc1* expression, when *tubb4b, rpl7l1l*, or *gapdh* were used as reference genes to analyze *kifc1* gene expression, it led to a significantly exaggerated or reduced expression level of *kifc1* in stage II testes (Figure 9). When *tubb4b* was used as reference genes, it also led to a significantly exaggerated expression level of *kifc1* in stage III testes (Figure 9). This result suggests that *tubb4b, rpl7l1l*, and *gapdh* are not ideal reference genes for studies involving testicular development.

## 4. Discussion

qPCR technology is a good method and has been widely used to detect the expression of target genes in tissues and during testes development to identify genes involved in testes development and spermatogenesis in animals [16,17]. In qPCR experiments, one or more housekeeping genes are commonly used as reference genes to normalize the expression of target genes [3,27,28]. However, housekeeping genes are not always expressed stably under all conditions. Some of them may not be appropriate for use as reference genes in qPCR analyses under specific conditions [4,5,6,7,31]. The use of an incorrect reference gene will undoubtedly lead to inaccurate conclusions. Therefore, it is necessary to screen for stably expressed reference genes under the specific experimental conditions used. In fishes, studies have documented the screening of reference genes [4,5,6,7,31], yet no such investigations have been specifically reported for suitable reference genes in *H. labeo*. In this study, we analyzed the expression stability of nine candidate reference genes in different tissues, as well as at different stages of testes development using RefFinder, geNorm, NormFinder, BestKeeper, and comparative delta-Ct methods. Our findings indicate that *eef1a* and *rps27* exhibit commendable stability of expression in different tissues, while *eef1a* and *actb* maintain a stable expression profile during testes development. Consequently, based on the stability of expression among candidate reference genes, *eef1a* is recommended as a reference gene for qPCR analysis in tissues and during testes development in *H. labeo* (the consistency observed in *kifc1* expression results when using eef1a as a single reference gene compared to analyses employing multiple reference genes further substantiates this recommendation). Similarly, the *eef1a* has also been recommended as a reference gene in *Monopterus albus* and *Puntius sophore* [4,6].

It has been suggested and widely accepted that employing multiple reference genes for normalizing gene expression data using the geometric mean method is essential to ensure accurate assessment of gene expression levels [28,30]. To determine the optimal number of reference genes required for quantification in tissues and during testes development in *H. labeo*, the geNorm algorithm was employed to analyze pairwise variation (Vn/Vn+1) between consecutive normalization factors (NFn and NFn+1) until reaching the point where only the last two genes remained under evaluation [30]. This analysis has already been accepted and applied in *Bos grunniens*, *Magang geese*, and *Oreochromis niloticus*, among others [5,18,19,31]. We determined that the computed pairwise variation values consistently fell below the recommended threshold of 0.15, as established by Vandesompele et al. [30], in tissue samples and during the developmental stages of the testes. These results suggest that two reference genes are adequate for accurate quantification in different tissue samples and testes at different stages of development. Based on the assessment of the stability of candidate reference gene expression, we propose the combined use of *eef1a* and *rps27* (their expression stability ranks among the top two) as reference genes in the analysis of gene expression in different tissue samples. Similarly, *eef1a* and *actb* are recommended as suitable reference genes during testes development.

KIFC1 protein, a member of the kinesin-14 family belonging to the C-kinesins, is capable of moving along microtubules (MTs) from the plus (+) end to the minus (−) end [32]. Apart from its function in material transportation, KIFC1 also plays a role in microtubule organization [33,34]. Furthermore, KIFC1 has been documented to hold crucial roles in regulating animal spermiogenesis [35]. In rats (*Rattus norvegicus*), the KIFC1 protein participates in vesicle transport to promote acrosome formation and interact with perinuclear microtubule and pucleoporin Nup62 to promote nucleus reshaping during spermiogenesis [36,37]. In *Octopus tankahkee*, Wang et al. [38] also discovered that KIFC1 colocalizes with perinuclear microtubules, facilitating the transition of the spermatid nucleus from a circular shape to a rod-like form. In teleost, there is no acrosome in the sperm, while nuclear concentration and reshaping exist during spermiogenesis [9,11]. The researchers also found that KIFC1 colocalizes with microtubules around the nucleus in Perciformes, including *Larimichthys crocea* and *L. polyactis* [16,17], implying the potential function of KIFC1 in the concentration and reshaping of spermatid nuclei in fish spermiogenesis. However, in other groups, such as Cyprinformes, the functions of KIFC1 were not reported. In this study, we analyze the expression of *kifc1* in different tissues with *eef1a* and *rps27* together as the reference genes and the expression of *kifc1* during testes development with *eef1a* and *actin* together as the reference genes. We found that *kifc1* was highly expressed in stage IV testes. Similar results were also found in *L. crocea* [16]. Stage IV in testes is the period of active spermiogenesis, where a large number of spermatids develop into sperm (Figure 3). The high expression of *kifc1* at this stage implies its important role in spermiogenesis. This result provides important evidence for the involvement of *kifc1* in the reshaping of spermatids in *H. labeo*. A potential mechanism is that *H. labeo* KIFC1 might also facilitate spermatid reshaping through interaction with perinuclear microtubules, as reported in *L. crocea* and *L. polyactis* [16,17].

We also analyzed *kifc1* expression using a single reference gene. We observed that in some tissues, when using *tubb4b*, *rpl7l1*, and *snrpd1* as the reference gene, the quantified expression level of *kifc1* exceeds its actual expression. On the contrary, with *gapdh* serving as the reference gene in certain tissues, the measured expression of *kifc1* falls short of its actual level. Furthermore, in stage II testes, when *tubb4b* or *rpl7l1* is used as the reference gene, the relative expression level detected of *kifc1* is higher than its actual expression level. On the contrary, when *gapdh* serves as the reference gene, the measured relative expression of *kifc1* is lower than its actual expression level. These results suggest that *tubb4b*, *rpl7l1*, and *gapdh* are not suitable as a reference gene in different tissues and during testes development in *H. labeo*. Similarly, Hu et al. [4] found that *gapdh* is unstable in expression and is not suitable as a reference gene in gonads of different developmental stages, as well as in other tissues of *M*. *albus*. The protein encoded by the *tubb4b* gene is the component that constitutes the microtubule. In rats, the microtubules are important proteins involved in the formation of manchettes to promote nuclear reshaping during spermiogenesis [39]. In fish, the microtubules are abundant in the spematid cytoplasm to promote spermiogenesis in *L. crocea*, and *L. polyactis* [16,17]. The involvement of the *tubb4b* gene in spermiogenesis may be an important reason for its unstable expression during testis development. The GAPDH protein constitutes a key enzyme in the glycolytic pathway, where it facilitates the initial step by effectively transforming D-glyceraldehyde 3-phosphate into 3-phospho-D-glycerate [40,41]. Glycolysis has been reported to be important for spermatogonial maintenance and self-renewal [42,43,44]. In stage II testes, where spermatogonia is predominant, it is likely that there exists a higher level of glycolysis to ensure spermatogonial maintenance and self-renewal, which may require increased expression of *gapdh* gene. Consequently, using *gapdh* as a reference gene could potentially underestimate the actual expression levels of the target genes in stage II testes. The *rpl7l1* may also be involved in testicular development, resulting in it being unsuitable as a reference gene for studies on testicular development.

## 5. Conclusions

In the study, we determined that *eef1a* is an appropriate reference gene when the 2^−ΔΔCt^ method is used for qPCR analysis in different tissues and during testicular development. Conversely, *tubb4b*, *gapdh*, and *rpl7l1* were found to be unsuitable as reference genes in both tissues and during testicular development. Furthermore, our results indicate that using the geometric mean of two reference genes, *eef1a* and *rps27*, is adequate for accurate quantification in tissue samples, while the combination of *eef1a* and *actb* suffices during testicular development stages. Therefore, we recommend the pairing of *eef1*a with *rps27* as reference genes for tissue qPCR analyses and *eef1a* with *actb* for qPCR analyses during testicular development.

Moreover, our investigation revealed that the *kifc1* gene displays high expression levels in stage IV testes, a highly active phase of spermiogenesis, suggesting a crucial role for *kifc1* in spermatid reshaping within *H. labeo*. Consequently, this research furnishes a solid foundation for studies involving qPCR experiments, especially those concentrating on testicular development, and contributes valuable insights into the functional role of *kifc1* in testicular development and spermatogenesis of *H. labeo*.

## Figures and Tables

**Table 1:** Full name of the candidate reference genes and their GenBank accession number.

Gene	GenBank Accession Number
glyceraldehyde-3-phosphate dehydrogenase (*gapdh*)	PP530450
beta-actin (*actb*)	PP530451
beta-tubulin (*tubb4b*)	PP530452
elongation factor 1-alpha (*eef1a*)	PP530453
40S ribosomal protein S27 (*rps27*)	PP530455
40S ribosomal protein S4 (*rps4*)	PP530454
18s rRNA small subunit ribosomal RNA (*18s*)	PP527010
small nuclear ribonucleoprotein D1 polypeptide (*snrpd1*)	PP530456
ribosomal protein L7-like protein 1 (*rpl7l1*)	PP530457
kinesin family member C1 (*kifc1*)	PP946169

**Table 2:** Primers and their PCR amplification efficiencies.

Primer Name	Sequence (5′ to 3′)	Dosage (µL) ^1^	Amplification Size (bp)	PCR Amplification Efficiency	Correlation Coefficients
*gapdh*F	CCGTGCTGCTATCCAGTCCAAGA	0.3	140	103.7%	0.990
*gapdh*R	TGCCGCCTTCTGCCTTAACCT	0.3			
*actb*F	ATGGTATCGTGATGGACTCTGGTGAT	0.6	162	101.4%	0.999
*actb*R	TGGTGGTGAAGCTGTAGCCTCTC	0.6			
*tubb4b*F	GCCGTATGTCCATGAAGGAGGTG	0.6	163	100.2%	0.999
*tubb4b*R	GCTGTGCTATTGCCGATGAAGGT	0.6			
*eef1a*F	ACTGCCACACTGCTCACATTGC	1.0	221	95.4%	0.997
*eef1a*R	AACAGCGACGGTCTGCCTCAT	1.0			
*rpl7l1*F	CACTCATAGAGCAGCATCTTGGACAA	0.3	125	97.9%	0.993
*rpl7l1*R	TGACAAATGGAACGGCAACAGGAA	0.3			
*rps27*F	GGAGAGGAGGAGGCACAAGAAGAA	0.5	146	94.8%	0.994
*rps27*R	GCACAGTTGAGCAACCGACACA	0.5			
*rps4*F	CGAGGACCGAAGAAGCATCTGAAG	0.5	232	102.1%	0.999
*rps4*R	CATCAGTGCGGACCTTGCCATC	0.5			
*18s*F	GGACACGGAAAGGATTGACAGATTGA	1.0	120	99.4%	0.999
*18s*R	CGGAGTCTCGTTCGTTATCGGAATG	1.0			
*snrpd1*F	AACCGTCACCATTGAGCTGAAGAAT	0.8	173	108.7%	1.000
*snrpd1*R	GCAGGATGAAGTAGCGGATGTTGT	0.8			
*kifc1*F	GCAGCGGGAAGACCTTTACTATGG	0.6	112	106.1%	0.999
*kifc1*R	CCTTGCTCTCGGAGTGCTTTGG	0.6			

^1^ The primer concentration is 10 µM.

## Data Availability

The original contributions presented in the study are included in the article/Supplementary Materials, further inquiries can be directed to the corresponding author.

## Supplementary Materials

The following supporting information can be downloaded at: https://www.mdpi.com/article/10.3390/ani14132006/s1, Figure S1: Specificity detection of the primers used in this study.
