# Supplementary material for: Screening of Reference Genes for Quantitative Real-Time PCR Analysis in Tissues and during Testis Development, and Application to Analyze the Expression of kifc1 in Hemibarbus labeo (Teleostei, Cypriniformes, Cyprinidae)
Source: Animals (Basel). 2024 Jul 7;14(13):2006. doi: 10.3390/ani14132006 (PMC11240434; doi:10.3390/ani14132006)
Supplement: Supplementary file 1 [file animals-14-02006-s001.zip › Figure S1.pdf]

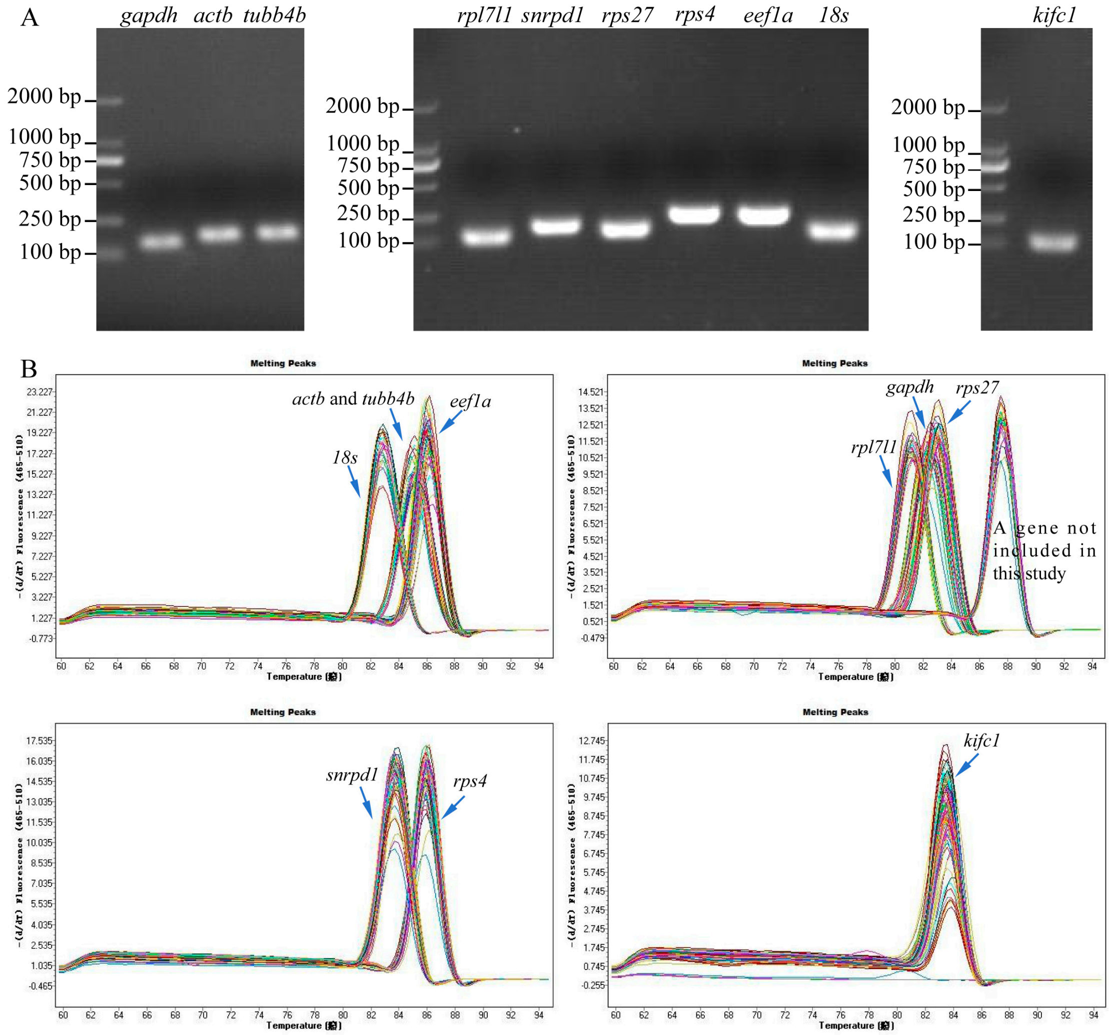

Figure S1. Specificity detection of the primers used in this study. (A) Agarose gel electrophoresis was used to analyze the primer specific. (B) Solubility curve was used to analyze the primer specific. The primers used to amplify the nine candidate reference genes and *kifc1* were specific.
